# Regulators, functions, and mechanotransduction pathways of matrix stiffness in hepatic disease

**DOI:** 10.3389/fphys.2023.1098129

**Published:** 2023-01-12

**Authors:** Ting Guo, Cindy Wantono, Yuyong Tan, Feihong Deng, Tianying Duan, Deliang Liu

**Affiliations:** ^1^ Department of Gastroenterology, The Second Xiangya Hospital of Central South University, Changsha, China; ^2^ Research Center of Digestive Disease, Central South University, Changsha, China

**Keywords:** liver fibrosis, extracellular matrix, matrix stiffness, mechanotransduction, mechanotherapy

## Abstract

The extracellular matrix (ECM) provides physical support and imparts significant biochemical and mechanical cues to cells. Matrix stiffening is a hallmark of liver fibrosis and is associated with many hepatic diseases, especially liver cirrhosis and carcinoma. Increased matrix stiffness is not only a consequence of liver fibrosis but is also recognized as an active driver in the progression of fibrotic hepatic disease. In this article, we provide a comprehensive view of the role of matrix stiffness in the pathological progression of hepatic disease. The regulators that modulate matrix stiffness including ECM components, MMPs, and crosslinking modifications are discussed. The latest advances of the research on the matrix mechanics in regulating intercellular signaling and cell phenotype are classified, especially for hepatic stellate cells, hepatocytes, and immunocytes. The molecular mechanism that sensing and transducing mechanical signaling is highlighted. The current progress of ECM stiffness’s role in hepatic cirrhosis and liver cancer is introduced and summarized. Finally, the recent trials targeting ECM stiffness for the treatment of liver disease are detailed.

## 1 Introduction

The extracellular matrix (ECM) is a cell-secreted extracellular microenvironment containing various components, such as collagen, fibronectin, laminin, and hyaluronic acid (Li et al., 2021). This extracellular microenvironment is crucial in hepatic functions and provides not only biochemical but also mechanical cues to influence cellular phenotype and behavior (Yeh et al., 2002; Desai et al., 2016). Increasing ECM stiffness is associated with liver pathological conditions including hepatic fibrosis resulted from viral hepatitis B and C, non-alcoholic fatty liver disease (NAFLD), autoimmune liver diseases, hereditary diseases such as Wilson’s disease, and liver cancer (Parola and Pinzani, 2019). The stiffness of ECM is typically reported as elastic modulus (E, also known as Young’s modulus) or shear modulus (G). Several mechanical test methods have been used to measure the ECM mechanical property, including atomic force microscopy, ultrasound elastography, magnetic resonance elastography, and fluorescent microscopy (Guo et al., 2022). The accumulated evidence revealed that ECM stiffening, a symbol of tissue fibrosis, is not merely an epiphenomenon of liver fibrosis, but can actively promote dysregulation of cellular function, leading to persistent and/or progressive liver fibrosis (Chen et al., 2019).

Here we aim to present an overview of the role of ECM stiffness in liver disease. We will start with summarizing the regulators of ECM stiffness in the liver mainly including ECM components, matrix metalloproteinase (MMPs), enzymatic or non-enzymatic crosslinking, and cell properties. We discuss the effects of matrix stiffness on cellular behavior and phenotype in various liver cell types, and the activity of key biomolecules (TGF-β). We review how cells sense ECM mechanical signals and the intercellular mechanotransduction pathways. Finally, we summarize the role of matrix stiffness in liver cirrhosis and hepatic cancer, and potential mechanotherapy. Collectively, this review reinforces the importance of matrix mechanics in hepatic fibrosis, and hopes to provide novel research directions and targets for hepatic disease.

## 2 Modulation of hepatic ECM stiffness

**FIGURE 1:**
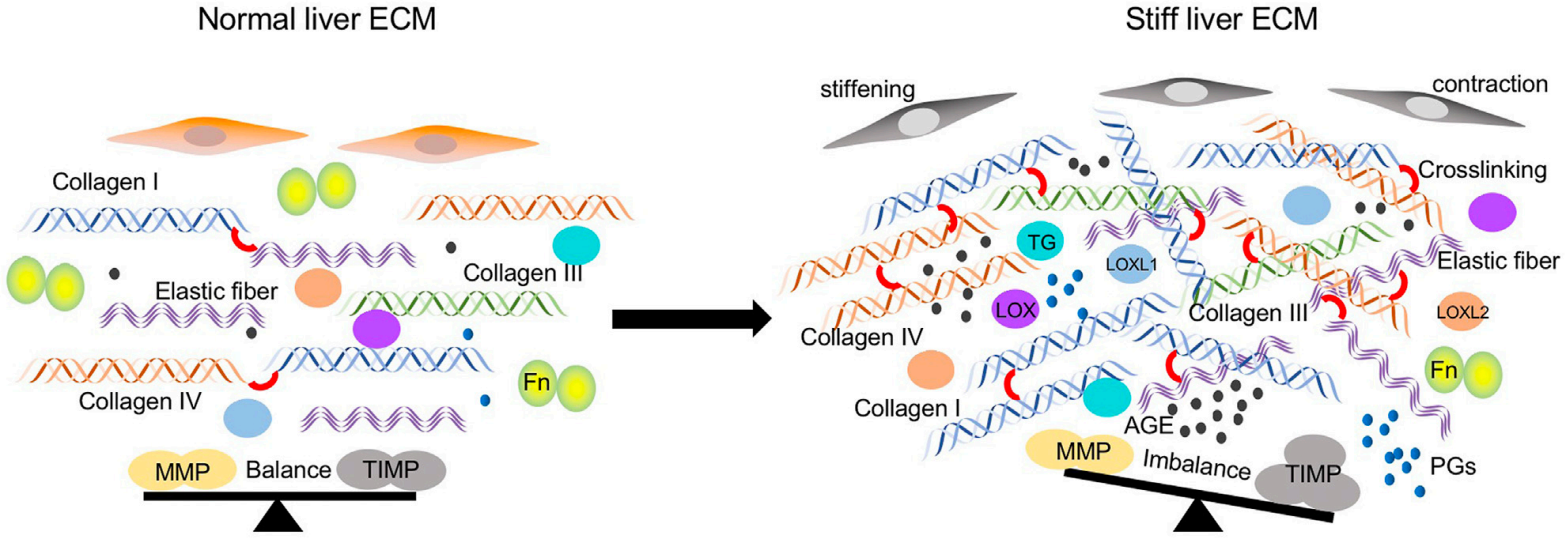
Regulators participated in ECM stiffening in the pathological lung tissues. ECM components including collagen type I, III, IV, and elastic fibers are significantly increased. Crosslinking between ECM components is elevated caused by increased crosslinking enzymes such as LOX family, TGs, and non-enzymatic AGEs modifications. The balance between MMPs and TIMPs is disturbed due to the increased expression of TIMPs and decreased MMPs. Cells stiffening and contraction also contribute to the matrix stiffness.

Many factors participate in promoting stiffness of the ECM in the pathological liver tissues (Figure 1), including the abnormal accumulation of ECM components such as collagen, fibronectin, and elastin, the dysregulated secretion of MMPs and TIMPs, and the excessive crosslinking. Moreover, the cell property also modulates the mechanics of the ECM.

### 2.1 ECM components and MMPs

During the fibrotic process, ECM components present an obvious change as the advanced fibrotic hepatic tissue contains approximately 6 times more ECM than the normal, including collagen type I, III, and IV, fibronectin, undulin, elastin, laminin, hyaluronan, and proteoglycans (Bataller and Brenner, 2005). Collagens are mainly produced by hepatic stellate cells and portal fibroblasts (Karsdal et al., 2020). In a carbon tetrachloride (CCl4) induced rat liver fibrosis model, the accumulation of total collagen increased as fibrosis progressed, which is 4.94% of the liver sections at 4 weeks, 8.25% at 6 weeks, and 9.11% at 8 weeks (Vassiliadis et al., 2011). Further measurements revealed that the liver matrix was significantly stiffer in regions approaching fibrillar collagen deposition and returned to near normal rigidity in areas remote from it within the same lobule (Desai et al., 2016). Collagen type I is the most common protein in the body and associated with stiffness across tissues generally (Swift et al., 2013). It’s the most excessive ECM components in fibrotic hepatic tissues (Kisseleva and Brenner, 2013), above, the massive deposition of collagen especially collagen type I is an essential contributing factor in the local stiffening of the ECM in progressive liver fibrosis. In addition to collagen accumulation, the stiffness of the ECM may also be influenced by the orientation of collagen. As Desai et al. (2016) found that the collagen bridges have more dense collagen but also more aligned collagen which can influence stiffness.

Elastic fiber is a compound consisted of amorphous elastin and fibrous fibrillin, which both are mainly made of smaller amino acids including glycine, valine, alanine, and proline (Kielty et al., 2002). It is actively synthesized by hepatic stellate cells and portal fibroblasts in diseased liver (Kanta 2016). In the CCl4-treated liver damage mice model, the increased stiffness of damaged liver ECM is accompanied with decreased elastic fibers. While in the diethoxycarbonyl dihydrocollidine (DDC)-treated livers, the expression of elastic fibers showed no alteration (Klaas et al., 2016). From the study conducted on patients with hepatitis C, the elastin increased from stage F2-F3 (Yasui et al., 2019). These studies indicate that elastic fibers may play a complex role in the stiffness of liver fibrosis, which may be associated with the causes of disease.

Fibronectin (Fn), mainly secreted and synthesized by endothelial cells, Kupffer cells, and hepatocytes, is a large dimeric glycoprotein existing in its insoluble form as a part of the ECM(Younesi and Parsian, 2019). The livers of Fn-null mice present extensive fibrosis, which is accompanied by elevated hepatic matrix rigidity and impaired liver functions. Mechanistically, mutant livers show increased TGF-β bioactivity and TGF-β-regulated lysyl oxidase (LOX) expression, which in turn promote ECM remodeling and stiffening (Iwasaki et al., 2016). These studies indicate a potential association between Fn-mediated control of TGF-β bioavailability and collagen fibril stiffness regulated by LOX.

Proteoglycans (PGs) are formed by glycosaminoglycans (GAGs) bound to a core protein, which can be a part of the ECM (Amendum et al., 2021). PGs have been demonstrated enhancement in remodeling liver tissues (Kimura et al., 2008). The amount of PGs increased significantly in stiffened liver tumors, especially perlecan and decorin (Kovalszky et al., 1993). The knockout of perlecan and decorin results in decreased matrix stiffness in murine model (Costell et al., 1999; Robinson et al., 2017).

MMPs and their inhibitors metalloproteinase tissue inhibitors (TIMPs) are normally in a dynamic balance to keep the expression and deposition levels of the ECM in the liver. While fibrosis occurs, their balance will be interrupted and the profibrogenic effects of TIMP-1 are thought to be mediated *via* preventing collagen degradation through inhibition of MMPs (Knittel et al., 2000), which changes mechanical properties of the liver matrix. Studies confirmed that increasing expression of TIPMs were detected in patients with liver injury and cirrhosis (Dechêne et al., 2010; Latronico et al., 2016). The imbalance secretion of MMP-9 and TIMPs mediated by rigid matrix perpetuate liver fibrosis (Lachowski et al., 2019). These finding shows that change of the deposition of ECM components in liver matrix, eventually altering the matrix stiffness of liver and affecting the process of hepatic fibrosis.

### 2.2 Enzymatic and non-enzymatic crosslinking

In addition to the promotion of matrix stiffness by the change of ECM components, the degree of cross-linking of the ECM affects the mechanical characteristics of the matrix.

Enzymatic cross-linking is major mediated by LOX, which increases the cross-linking of collagens and subsequently elevate matrix stiffness (Barker et al., 2012). The LOX family, comprising LOX and four lysyl oxidase-like proteins (LOXL1-4), are secreted copper-dependent amine oxidases, which catalyze the oxidative deamination of primary amine groups into reactive aldehyde condense with other aldehydes or ε-amino groups of selected lysine and hydroxylysine residues to form covalent intra- and inter-molecular crosslinks (Shah et al., 1993; Eekhoff et al., 2018). The stellate cells and portal fibroblasts were the major cellular sources of LOXs in CCl4-induced model of liver fibrosis, and LOX-mediated deoxypyridinoline and pyridinoline cross-links increased correspondingly with liver stiffness (Perepelyuk et al., 2013). Previous studies found that expression of LOX, LOXL1, and LOXL2 are increased in patients with liver fibrosis (Vadasz et al., 2005; Chen et al., 2020). The inhibition of LOX suppressed the cross-linking of collagen and attenuated CCl4-induced advanced hepatic fibrosis (Liu et al., 2016). Besides the expression, the increased activity of LOX family members are also observed in sera of patients with liver fibrosis cirrhosis (Mesarwi et al., 2015). Studies have shown that higher expression of LOX is a predictor of progression and poor prognosis in patients with liver cancers (Lin et al., 2020; Sun et al., 2022). In addition to collagen, LOX1 could also increase the cross-linking of elastin and promote the liver cirrhosis (Zhao et al., 2018).

Transglutaminase (TGs) are calcium-dependent enzymes that catalyze covalent crosslinking between the γ-carboxy-amine group of glutamine residues and the ε-amino group of lysine residues, generating ε-(γ-glutamyl)lysine isopeptide crosslinks (Griffin et al., 2002). The expression of TG is upregulated in experimental liver fibrogenesis (Mirza et al., 1997; Chen et al., 2008). Further study indicated that TG-mediated cross-linking occurs in the liver extracellular matrix during the early, inflammatory, stage of liver fibrosis, whereas cross-linking by pyridinoline occurs mostly later in the fibrotic process^8^. The inhibition of TG has been demonstrated to ameliorate liver fibrogenesis in animal models (Qiu et al., 2007; Tang et al., 2014).

Advanced glycation end products (AGEs) are non-enzymatic modifications of protein crosslinking. It differs from enzymatic process as AGEs can form crosslinks throughout the collagen molecule not only at the N- and C-terminal ends of the molecule (Brownlee et al., 1988). Accumulation of AGEs induces abnormal matrix crosslinking that ultimately leads to a progressive increase in tissue stiffness. The upregulation of AGEs accelerates liver injury and fibrosis (Goodwin et al., 2013). In a rat liver fibrosis model, the contents of AGEs in their blood sera and liver homogenates were increased in the late phase, and it could reflect the fibrosis degree of the rat livers (Gao et al., 2006). In patients with hepatitis C-related cirrhosis, AGEs are powerful parameters associated with the HCC occurrence (Abdel-Razik et al., 2021). These data suggest that the AGEs accumulation may take a function in the late stage of liver fibrosis and the inhibition of AGEs may provide a novel target for late patients with cirrhosis.

### 2.3 Cell stiffening and contraction contributes to the stiffness of liver

The cells’ physical properties and interaction with microenvironment could alter the stiffness of the ECM. In the *in vitro* model for liver cirrhosis, the stiffness of human hepatoma-derived HepG2 cells was significant increased after treatment with three known induction factors (collagen substrates, alcohol, and CCl4) (Sun et al., 2014). The lung with idiopathic pulmonary fibrosis remains only 44% of the original lung stiffness after decellularized treatment (Booth et al., 2012). These evidence suggest cell stiffness modulates the mechanical property of ECM. Active microrheology (AMR) analysis showed that cells grown in 3D type 1 collagen gels modulate the mechanical microenvironment in a cellular contraction-dependent manner. Inhibition of cellular contractility instantaneously softens the pericellular space and reduces stiffness heterogeneity (Keating et al., 2017). Rocky et al. further demonstrated that promotion of contraction of hepatic stellate cells contribute to the cirrhotic liver (Rockey and Weisiger, 1996). Furthermore, proteoglycans in the ECM can influence the cell contraction based alignment (Chen et al. Sci Reports. 2020). Together, these data suggest a central role for cellular physical property and contractility modifying the ECM stiffness.

## 3 Functions of the matrix mechanics

### 3.1 Regulation of phenotype and behavior of various cell types

#### 3.1.1 Hepatic stellate cells

Hepatic stellate cells (HSCs)are major cellular sources of myofibroblasts driving liver fibrogenesis. In the fibrogenic liver, quiescent HSCs transdifferentiate into proliferative, migratory, and contractile myofibroblasts, manifesting pro-fibrogenic transcriptional and secretory properties, and secrete ECM molecules (Higashi et al., 2017). HSC became progressively myofibroblastic as substrate stiffness increased on all coating matrices (Olsen et al., 2011). It has been shown that the stiffening of the ECM promotes HSCs activation to myofibroblasts through CD36-AKT-E2F3 mechanosignaling pathway, and these HSCs cultured on stiff matrices express high levels of α-smooth muscle actin (αSMA) (Liu et al., 2021). Wells et al. (2005) further suggested the interplay between matrix stiffness and TGF-β promotes HSCs differentiation through a two-step process, as the mechanical stiffness promotes the αSMA expression and TGF-β signaling affects the formation of focal adhesions and organization of stress fibers. Also, stiff matrices promote HSCs secretion of fibronectin and collagen, which could be inhibited by FHL2 knockdown (Kostallari et al., 2022).

#### 3.1.2 Hepatocyte

Hepatocytes are the main parenchymal cells and functional units of the liver. Previous studies have shown that hepatocytes contributed to the process of liver fibrosis through changing migratory behavior and biological functions. Hepatocytes are sensitive to substrate stiffness (Xia et al., 2018). Tingting et al. demonstrated that stiff matrix promoted hepatocellular migration through enhancing the formation of actin- and tubulin-rich structures, including the filopodia and lamellipodia. Moreover, hepatocytes cultured on stiff substrate showed increased apoptosis (Xia et al., 2020). Thus, matrix stiffness could change the behavior and phenotype of hepatocytes. Besides that, stiffer matrix downregulates the expressions of albumin, CYP450 reductase, and HNF4α collectively (Li et al., 2021; Monckton et al., 2021), and the HNF4α decrease is closely associated with stiffer substrates-induced hepatocytes proliferation (Schrader et al., 2011). The mechanotransduction in primary hepatocytes is transferred through focal adhesion kinase and downstream Rho/Rho-associated protein kinase pathway (Desai et al., 2016). Several key drug transporter genes (NTCP, UGT1A1, and GSTM-2) were downregulated in hepatocytes cultured on stiff matrix, which helps illustrate the poor prognosis of liver cirrhosis and carcinoma (Natarajan et al., 2015). The matrix stiffness-induced hepatocyte differentiation is mediated with ERK and ROCK (Kourouklis et al., 2016). Above all, matrix stiffness mediates various functions of hepatocytes including migration, proliferation, and differentiation.

#### 3.1.3 Immunocyte

In hepatocellular carcinoma (HCC) tissues, macrophage showed an M2 polarization, and the *in vitro* study confirmed that a stiff matrix could strengthen the M2 polarization and induce LOXL2 expression through integrin β5-FAK-MEK1/2-ERK1/2 pathway (Xing et al., 2021). This study helps clarify the underlying mechanism of matrix stiffness-induced premetastatic niche formation in HCC. Interfering with collagen stabilization reduces ECM content and tumor stiffness, leading to improved T cell migration and increased efficacy of anti-PD-1 blockade in the cholangiocarcinoma model (Nicolas-Boluda et al., 2021). All these data suggest ECM stiffness’s role in mediating the function of immunocytes, which provide new insight into interfering with hepatic disease.

### 3.2 Regulation of biomolecule activity

TGF-β1, a 25-kDa homodimeric protein, has been involved in hepatic fibrogenesis, regulation of liver cell growth, tumor development, and induction of hepatocellular apoptosis (Gressner et al., 1997; Flisiak et al., 2000). The TGF-β1-promoted HSCs activation is a dominant signal in liver fibrosis (Guvendiren et al., 2014). Increased TGF-β1 expression has been found in patients with hepatic fibrosis (Li et al., 2015; Kotsiri et al., 2016). TGF-β1 was secreted as a large latent complex (LLC), consisting of TGF-β1, latency-associated pro-peptide (LAP), and the latent TGF-β1 binding protein (LTBP-1). LTBP-1 is an ECM protein binding to several other ECM components, including fibrillin-1, Fn, and vitronectin, thereby depositing latent TGF-β1 within the ECM(Annes et al., 2003; Robertson et al., 2015). Cells contraction activates latent TGF-β1 from a preformed ECM, and this TGF-β1 activation increases with augmented matrix stiffness (Wipff et al., 2007; Giacomini et al., 2012). Together, the efficiency of latent TGF-β1 activation depends on the mechanical properties of the ECM.

## 4 Sensing and mechanotransduction

**FIGURE 2:**
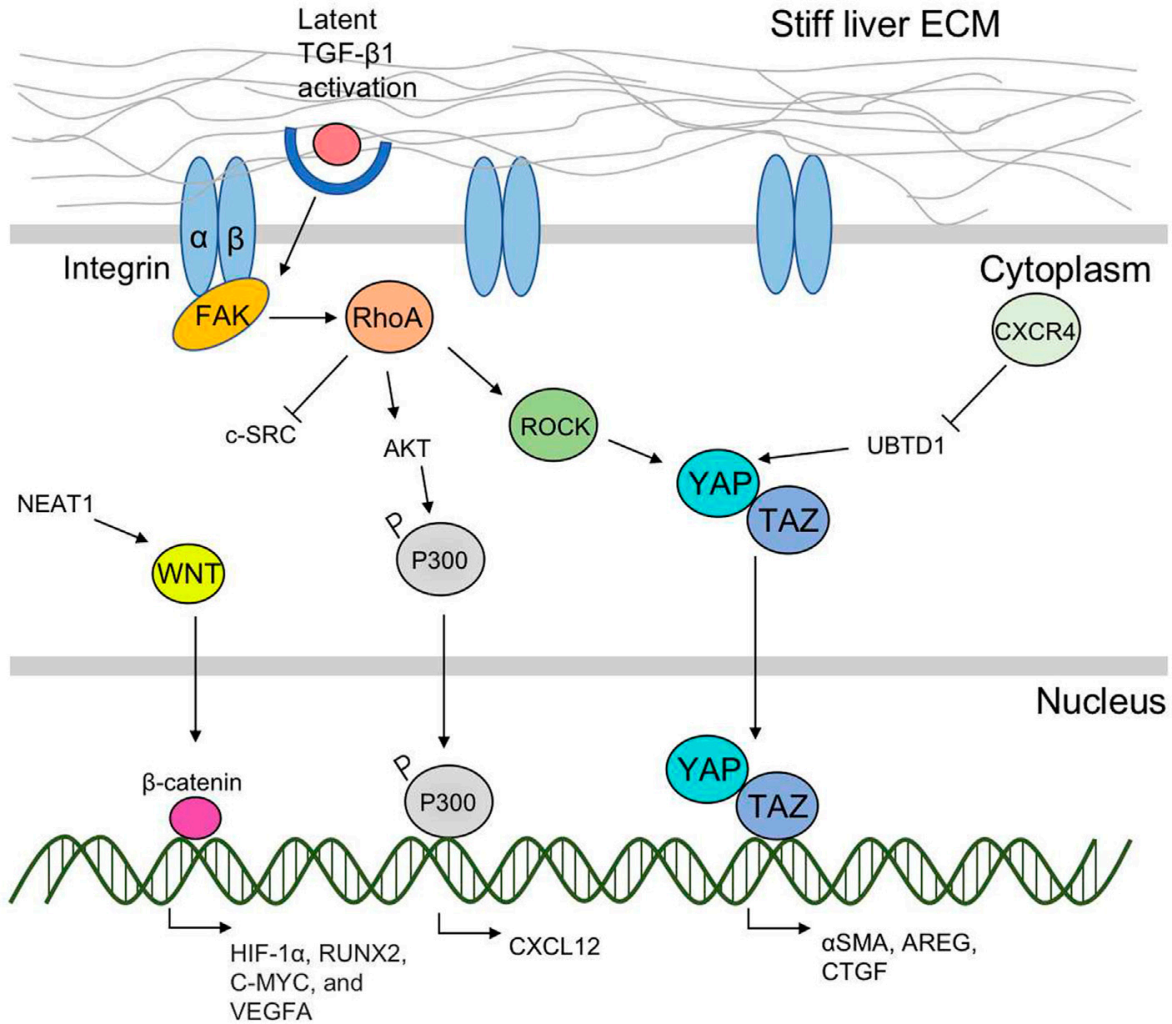
A scheme of liver ECM stiffness-regulated sensing and mechanotransduction pathways. Liver ECM-derived mechanical cues are mainly sensed by integrin-based FAs located on the cell surface. The intercellular mechanotransduction pathways including the RhoA/ROCK and the YAP/TAZ signaling ways are further activated and transfer the mechanical cues into nucleus to target profibrotic genes. RhoA/ROCK interacts with YAP/TAZ, and promotes their nuclear translocation. RhoA activates AKT, promotes P300 phosphorylation, and CXCL 12 expression. Matrix stiffness-induced CXCR4 expression inhibits UBTD1 and activates YAP. The nuclear translocation of YAP/TAZ promotes expression of α-SMA, AREG, and CTGF. Other mechanotransduction pathway includes NEAT1-WNT/β-catenin pathway.

Integrin-based focal adhesion (FA) complexes are the major cellular components responsible for detecting mechanical signals produced by the ECM(Discher et al., 2005). In mouse fibrotic livers, the stiffened matrix promotes integrin clustering and then activates the kinase activity of focal adhesion kinase (FAK). The genetic knockout of FAK in hepatocytes has been shown to protect mice from inflammation and fibrosis (Santos and Lagares, 2018). The intercellular mechanotransduction pathway mainly includes the RhoA/ROCK and the YAP/TAZ signaling way (Figure 2).

### 4.1 The RhoA/ROCK pathway

The small Rho GTPase RhoA/Rho kinase (ROCK)-mediated mechanotransduction pathway is activated by matrix stiffness (Ohashi et al., 2017). The substrate stiffness could directly activate FAK, and induce the downstream cascades of intracellular signals of the RhoA/ROCK pathway (Peng et al., 2019). Specifically, there are two ROCK isoforms (ROCK1 and ROCK2), and the matrix stiffness-activated ROCK isoforms differentially regulated the pathways of RhoA/ROCK1/p-MLC and RhoA/ROCK2/p-cofilin in a coordinate fashion to modulate cell motility (Peng et al., 2019). Recently, Dou et al. demonstrated that matrix stiffening can activate AKT through inducing phosphorylation and accumulation of p300 to the nucleus of HSCs and promote cancer metastasis by elevating the expression of paracrine factors such as CXCL12. In this process, the RhoA pathway of human HSCs was activated by the matrix stiffness from 0.4 kPa to 25.6 kPa and accelerated the downstream nuclear translocation of p300 leading to HSCs activation, which suggests that RhoA is required of substrate stiffness-promoted phosphorylation of p300 (Dou et al., 2018). Inhibition of the RhoA/ROCK pathway has been demonstrated effective in attenuating fibrogenesis in several animal hepatic fibrosis models (Xie et al., 2018; Lai et al., 2019).

### 4.2 The YAP/TAZ pathway

Yes-associated protein (YAP) and its close paralog transcriptional coactivator with PDZ-binding motif (TAZ) are activated in HSCs in response to matrix stiffening (Caliari et al., 2016). Immunohistochemical examination of fibrotic liver tissues from patients with hepatitis C found that the myofibroblasts in the fibrotic region with strong nuclear staining of YAP and TAZ (Mannaerts et al., 2015). The stiff substrates promote YAP/TAZ nuclear translocation from the cytoplasm (Dupont et al., 2011), and induce the expression of profibrotic genes and increase the expression of α-SMA and excessive matrix accumulation (Panciera et al., 2017). The inhibition of YAP by verteporfin reduced HSC activation and fibrogenesis (Zhang et al., 2016). RhoA/ROCK signaling is crucial for YAP/TAZ activation, which has been experimentally demonstrated in various systems, either genetically or by using inhibitors (Panciera et al., 2017). Though evidence showed that YAP/TAZ activity in other liver cells including hepatocytes, Kupffer cells and liver sinusoidal endothelial cells are increased and promotes inflammation and fibrosis in multiple models of non-alcoholic steatohepatitis and chemical-induced liver fibrosis, the relationship between matrix stiffness and its influence on these cells needs further elucidation (Zhang et al., 2016). Mechanistic studies suggested that CXCR4 is a critical intracellular signal transducer that relays matrix stiffness signals to inhibit ubiquitin domain-containing protein 1 (UBTD1), which then activates YAP signaling pathway (Yang et al., 2020). And the TAZ could further target gene *AREG* and *CTGF* to promote liver fibrogenesis (Yang et al., 2012).

## 5 Matrix stiffness in the pathogenesis of liver disease

### 5.1 Liver cirrhosis

The pathological stage of hepatic fibrosis is generally divided into 5 grades: F0 is non-hepatic fibrosis, F1 is mild hepatic fibrosis, F2 is moderate hepatic fibrosis, F3 is severe hepatic fibrosis, and F4 is cirrhosis. Golo Petzold examined patients with healthy liver or cirrhosis with 2-dimensional shear wave elastography and found that the normal liver stiffness to be 4.93 ± 0.83 kPa and F4 cirrhotic liver to be 13.29 ± 3.27 kPa (Petzold et al., 2019). The higher liver stiffness indicates a higher possibility of clinical complications and a worse prognosis in patients with advanced chronic liver disease (Vergniol et al., 2011; Singh et al., 2013). Even a study found that the increased stiffness precedes fibrosis and potentially promotes fibrotic pathogenesis in an animal liver injury model. This early change appears to result from matrix cross-linking mediated by an imbalanced LOX family (Georges et al., 2007). Cell types including hepatocytes, hepatic stellate cells, and liver sinusoidal endothelial cells, isolated from rats with cirrhosis and cultured on a rigid matrix, presented an altered nuclear morphology compared with those on soft matrix, which suggests mechanotransduction could potentially influence the phenotype of various liver cells and accelerate cirrhosis process (Guixé-Muntet et al., 2020). The expression of mechanotransducer including TAZ and serum osteopontin (OPN) is associated with increased liver stiffness in patients with liver cirrhosis, especially in autoimmune- and alcohol-related cirrhosis (Khajehahmadi et al., 2020). The nuclear translocation of TAZ could target genes *AREG* and *CTGF,* and the overexpression of the two genes promotes the liver cirrhosis process (Mitani et al., 2009; Yang et al., 2012; Mohagheghi et al., 2019). In liver cirrhosis, the mechanical transduction pathway RhoA/ROCK was activated and led to increased vascular contractility and portal pressure (Hennenberg et al., 2006; Trebicka et al., 2007), moreover, the matrix stiffness-induced RhoA overexpression also interacts with the cytosolic tyrosine kinase c-SRC and decreases the c-SRC activity to activate HSC to mediate liver cirrhosis (Görtzen et al., 2015). The expression of TIMP-1 is increased in hepatic cirrhosis patients, which is partly caused by matrix stiffness-induced HSC’s elevated exocytosis and secretion of TIMP-1 in a caveolin-1-and dynamin-2-dependent manner (Lachowski et al., 2022).

Portal hypertension is a complication for patients with compensated advanced chronic liver disease or decompensated cirrhosis. Liver stiffness measurements (LSM) offer valuable alternatives to detect and monitor portal hypertension. The thresholds of liver stiffness>15 kPa is highly suggestive of advanced chronic liver disease, and>20–25 kPa indicates a high likelihood of clinically significant portal hypertension (Reiberger 2022). Moreover, LSM has become an important instrument to assess the clinical course of portal hypertension, as patients with baseline LSM >14 kPa have a worse prognosis regarding both development of complications or survival (Stefanescu and Procopet, 2014).

### 5.2 Liver cancer

Increased matrix stiffness is a mechanical feature of solid tumors, including liver cancer. HCC cells cultured on stiff matrix showed a significantly higher proliferative index than that on soft matrix (Schrader et al., 2011). A rigid matrix promotes proliferation of HCC cells by stimulating expression of integrin β1, activating the PI3K/Akt pathway, and upregulating VEGF expression (Dong et al., 2014). ECM-derived mechanical signals promote HCC cell invasion and metastasis, which is also mediated by integrin β1 (Zhao et al., 2010). Moreover, higher stiffness-stimulated HCC cells exhibited chemotherapeutic resistance to cisplatin (Schrader et al., 2011), paclitaxel, 5-FU (Liu et al., 2015), weakened oxaliplatin-induced apoptosis (You et al., 2016), and attenuated metformin-inhibited invasion and metastasis (Gao et al., 2020). Studies above indicate that matrix stiffness accelerated the pathogenesis of liver cancer through mediating HCC cells phenotype. Epithelial-mesenchymal transition (EMT) is a phenomenon that HCC cells presents fibroblast-like morphology, cytoskeleton remodeling, protrusive and invasive pseudopodial structure, similar to morphology and great migration capability, which promotes the development of liver cancer (Giannelli et al., 2016). High stiffness could induce EMT in HCC cell and is related to overexpression of Snail, which is converged by three signaling pathways including S100A11 membrane translocation, eIF4E phosphorylation, and TGF-β1 autocrine (Dong et al., 2019). Besides that, the activated NEAT1-WNT/β-catenin pathway is confirmed in substrate stiffness-regulated EMT in HCC cells (Dong et al., 2019). Liver cancer cells with stemness properties (LCSCs) comprise a small portion of HCC cells. This small proportion of HCC cells has great self-renewal and metastatic potential. Research has shown that the stiffnesses of the liver tumor invasive front and core are different, as the core is softer than the invasive front (Sun et al., 2021). Boren Tian et al. demonstrated that a soft matrix increases the stemness of HCC cells, which may be beneficial to maintain dormancy and adaptation to the surrounding environment and then initiate tumorigenesis when the microenvironment is suitable (Tian et al., 2019). Furthermore, the soft spot-enhanced CSCs stemness is related to drug resistance and HCC metastasis (Ng et al., 2021).

Activated HSCs in the liver microenvironment regulate HCC growth by paracrine mechanisms, including secreting growth factors, ECM, and cytokines (Zhang and Friedman, 2012). The mechanical stimulation of ECM can induce HSCs to differentiate into cancer-associated fibroblasts (CAFs) (Yeung et al., 2005). Changwei Dou et al. demonstrated that substrate stiffness-activated HSCs through a RhoA-AKT-p300 signaling pathway (Dou et al., 2018). The other study indicated that stiffness-activated CD36-AKT-E2F3 signaling also takes a role in the HSCs transdifferentiation to CAFs by FGFR1-mediated PI3K/AKT and MEK/ERK signaling (Liu et al., 2021). All above suggest matrix stiffness-promoted HSCs activation is enrolled in HCC growth and metastasis.

## 6 Measurements of liver matrix stiffness

Matrix mechanical properties are typically reported as elastic modulus (E, also known as Young’s modulus) or shear modulus (G). There is an equation between elastic modulus and shear modulus, and the elastic modulus is approximately three times the shear modulus for isotropic and incompressible materials. Several mechanical test methods have been used to measure the matrix stiffness, including elastography and atomic force microscopy (AFM) methodology (Sigrist et al., 2017; Ojha et al., 2022).

### 6.1 Elastography

Ultrasound elastography (USE) is an imaging technology sensitive to organ mechanical property that was first introduced in the 1990s (Gennisson et al., 2013). Shear wave imaging (SWI) employs a dynamic stress to generate shear waves in the parallel or perpendicular dimensions. There are currently three technical approaches for SWI: 1) point shear wave elastography (pSWE), 2) 1 dimensional transient elastography (1D-TE), and 3) 2 dimensional shear wave elastography (2D-SWE) (Barr et al., 2015). pSWE can be performed on conventional US machine but it could be only stressed in a single focal location. 1D-TE can estimate the stiffness along ultrasonic A-line, but it is not user adjustable and no image-guided. 2D-SWE is the newest SWI method which can test stiffness in a rapid speed in multiple focal zones (Ferraioli et al., 2015). However, there are some common technical limitations for SWI, such as shadowing, reverberation, clutter artifacts, decreased ultrasound signal for deep tissues, or the operator-dependent nature of free-hand ultrasound system (Palmeri and Nightingale, 2011).

Magnetic resonance elastography (MRE) uses a modified phase-contrast imaging sequence to detect propagating shear waves within the liver. It provides parameters with high sensitivity to elasticity, viscosity, and poroelastic properties for the evaluation of structural variations in livers at multiple scales (Singh et al., 2016). MRE has been demonstrated a highly accurate non-invasive diagnostic method to detect and monitor various liver pathophysiologic states (Li et al., 2020). MRE is limited by long operating times, high costs, difficulty in distinguishing tissue stiffening between fibrosis and inflammation or altered perfusion, such as portal venous hypertension in cirrhosis (Salameh et al., 2009).

### 6.2 AFM

One of the major drawbacks of common liver stiffness measurements including USE and MRE is that they do not provide cellular-level resolution of stiffness heterogeneity in the liver (Ojha et al., 2022). During the liver disease development, areas containing rich collagens show higher stiffness compared to the surrounding parenchyma (Calò et al., 2020). Thus, changes in local rigidity need to be characterized on a microscopic level to better understand the progression of fibrosis. AFM could detect the liver mechanical properties with high resolution and high force sensitivity. AFM indents the tissue surface with the tip of a cantilever, and causes deformation at the microscopic or nanoscopic level according to the geometric shape and size of the tip. The force response of the tested sample to the applied strain is then calculated as the deflection in the cantilever (Binnig et al., 1986). The force displacement curve is collected from the approach and retraction of the cantilever, which can be fitted with an appropriate contact mechanical model to evaluate the local stiffness of the sample. AFM can also provide topographic information about specific features in the sample, such as the structure of collagen (Amabili et al., 2021). Moreover, AFM has also been used *in vitro* to determine the stiffness of cells and extracellular protein scaffolds (Norman et al., 2021).

## 7 Mechanotherapy

As the importance of matrix stiffness in mediating the liver fibrotic process, targeting the liver mechanics is a promising therapy to prevent or even reverse liver fibrosis. There are two main methods to interrupt the pathogenesis including decreasing the matrix stiffness directly and inhibiting the mechanotransduction pathways.

To reduce the matrix stiffness, targeting the LOXL2-induced crosslinking through anti-LOXL2 antibody AB0023 has been demonstrated effective in reliving liver fibrosis in animal models (Ikenaga et al., 2017). Another study confirmed that silencing LOXL1 can treat the progression of cirrhosis by reducing the crosslinking of elastin in CCl4-induced fibrosis mice models (Zhao et al., 2018). These studies indicated that inhibition of the LOX family has the potential to treat hepatic fibrosis. However, a phase II clinical trial conducted in IPF patients showed that the LOXL2-specific antibody Simtuzumab failed to improve progression-free survival (Raghu et al., 2017). This result suggests crosslinking and matrix stiffness involve complex mechanism in mediating organ fibrosis and needs further investigation in patients with liver fibrosis.

Fasudil is a clinically approved small-molecule inhibitor of ROCK. Treatment with Fasudil significantly attenuated the activation of ROCK and alleviates hepatic fibrosis in diabetic rats (Zhou et al., 2014; Xie et al., 2018). Other studies have shown that selective ROCK inhibitor Y27632 suppressed the Rho/ROCK pathway and attenuates α-SMA expression to prevent hepatic fibrosis in animal models (Tada et al., 2001). It would be promising to see whether targeting Rho/ROCK pathway could be effective for the treatment of patients with liver fibrosis.

YAP/TAZ pathway is activated in liver fibrosis, and the suppression of YAP levels by transfecting siRNAs or pharmacological inhibition of YAP by verteporfin can effectively decrease the activation of HSCs *in vitro* and can alleviate the fibrogenesis in an animal model with liver fibrosis (Mannaerts et al., 2015). Omega-3 polyunsaturated fatty acids (ω-3 PUFAs), such as docosahexaenoic acid (DHA) and eicosapentaenoic acid (EPA), demonstrate effectiveness in preventing liver fibrosis by targeting YAP to decrease the levels of pro-fibrogenic genes in HSCs and fibrotic liver (Zhang et al., 2016). These findings reveal that targeting the nuclear transcription of YAP/TAZ in cells represents a potential strategy for the treatments of fibrotic liver diseases.

## 8 Conclusion and future perspectives

The mechanical property of the ECM affects the phenotype and function of various liver cell types and is associated with hepatic disease including cirrhosis and carcinoma. Currently, the knowledge of mechanosensing and intercellular mechanotransduction mechanisms remains limited. A better understanding of how hepatic cells sense matrix stiffness and translate matrix mechanical cues into the cellular responses can provide us with more opportunities to develop accurate drugs or inhibitors targeting altered ECM mechanics in liver diseases. Though some intervention of matrix stiffness has achieved initial effects *in vitro* or animal models, there is still a lot of work to do to further verify their efficacy.
